# Variations in peak nasal inspiratory flow among healthy students after using saline solutions^☆^

**DOI:** 10.1016/j.bjorl.2015.03.012

**Published:** 2015-09-10

**Authors:** Jaime Olbrich Neto, Sandra Regina Leite Rosa Olbrich, Natália Leite Rosa Mori, Ana Elisa de Oliveira, José Eduardo Corrente

**Affiliations:** a Department of Pediatrics, Faculdade de Medicina de Botucatu, Universidade Estadual Paulista (UNESP), Botucatu, SP, Brazil; b Department of Nursing, Faculdade de Medicina de Botucatu, Universidade Estadual Paulista (UNESP), Botucatu, SP, Brazil; c Public Health, Faculdade de Medicina de Botucatu, Universidade Estadual Paulista (UNESP), Botucatu, SP, Brazil; d Department of Biostatistics, Instituto de Biociências de Botucatu, Universidade Estadual Paulista (UNESP), Botucatu, SP, Brazil

**Keywords:** Children, Peak nasal inspiratory flow, Nasal hygiene, Saline, Crianças, Pico de fluxo inspiratório nasal, Higiene nasal, Salina

## Abstract

**Introduction:**

Nasal hygiene with saline solutions has been shown to relieve congestion, reduce the thickening of the mucus and keep nasal cavity clean and moist.

**Objective:**

Evaluating whether saline solutions improve nasal inspiratory flow among healthy children.

**Methods:**

Students between 8 and 11 years of age underwent 6 procedures with saline solutions at different concentrations. The peak nasal inspiratory flow was measured before and 30 min after each procedure. Statistical analysis was performed by means of *t* test, analysis of variance, and Tukey's test, considering *p* < 0.05.

**Results:**

We evaluated 124 children at all stages. There were differences on the way a same concentration was used. There was no difference between 0.9% saline solution and 3% saline solution by using a syringe.

**Conclusion:**

The 3% saline solution had higher averages of peak nasal inspiratory flow, but it was not significantly higher than the 0.9% saline solution. It is important to offer various options to patients.

## Resumo

**Introdução:**

A higiene nasal com soluções salinas tem sido indicada para aliviar a congestão, reduzir o espessamento do muco e manter a cavidade nasal limpa e úmida.

**Objetivo:**

Avaliar se as soluções salinas melhoram o fluxo inspiratório nasal entre crianças sadias.

**Método:**

Escolares com idades entre 8 e 11 anos foram submetidos a 6 procedimentos com soluções salinas em diferentes concentrações. O pico de fluxo inspiratório nasal foi medido antes e 30 minutos após cada procedimento. A análise estatística foi realizada por meio do teste *t*, análise de variância e teste de Tukey, considerando *p* < 0,05.

**Resultados:**

Foram avaliadas 124 crianças em todas as etapas. Houve diferenças quanto à forma de uso de uma mesma concentração. Não houve diferença entre solução salina a 0,9% e solução salina a 3% por meio de seringa.

**Conclusões:**

A solução salina a 3% obteve maiores médias do pico de fluxo inspiratório nasal, porém não foi significativamente superior à solução salina a 0,9%. É importante oferecer diferentes opções aos pacientes.

## Introduction

Nasal hygiene has been shown to relieve congestion, reduce the viscosity of mucus and keep nasal cavity clean and moist. Nasal breathing is the only physiological type of breathing in humans, and is considered mandatory, although substituting mouth breathing is compatible with life.

The mucociliary layer, which covers the nostrils, actively participates in respiratory homeostasis through ciliary function, mucus secretion and the release of inflammatory mediators.^1^, ^2^, ^3^, ^4^ The maintenance of integrity of the respiratory mucosa is essential for the airways to fulfill their role; this can justify the use of external media, such as sprays, lavage and irrigation of the nasal cavity to promote or facilitate nasal hygiene.^2^, ^3^, ^4^, ^5^ The use of saline solutions seems to facilitate the transport of mucus, particles, irritants and microorganisms toward nasopharynx, probably by direct physical action and by increasing ciliary beating, which is reduced during inflammatory processes. In patients with chronic sinusitis, Ural et al. observed a reduction in mucociliary clearance with the saccharine clearance test. Min et al.,^6^ conducted an experimental study in which animals were submitted to staphylococcal toxin in different concentrations, and observed a reduction in the speed of ciliary beating and the development of an inflammatory infiltrate in rabbit maxillary sinus mucosa.

In children, the nose is narrower than in adults, and cold, pollution and allergic or infectious processes easily clog the nostrils. In children, nasal hygiene can and should be done in a natural and physiological way, at any time of day, in the morning and at bedtime. Greater frequency should be considered when the child stays in an indoor environment with air conditioning, in periods of low air humidity, and during allergic or infectious inflammatory processes. Nasal hygiene complements basic therapies and promotes normal mucosal function. The benefits of nasal application of saline solutions have been demonstrated for decades by several authors. In a review article, Khianey et al.^7^ concluded that the benefit is small but there are few side effects and it is well tolerated, a fact also confirmed by Jeffe et al.,^8^ who studied the tolerance and use of saline solutions in 61 children. The use of saline solutions as a complementary therapy or as treatment has not been established yet. Fashner et al.^9^ suggested the use of these solutions for 3 weeks for common cold cases, and felt they could be used for 9 weeks as a preventive measure. Hermelingmeier et al.,^10^ in a review article, concluded that saline solutions should be used as a complementary therapy.

Compared to isotonic solutions, the use of solutions with higher concentrations of sodium has promoted better mucociliary function responses in patients with chronic rhinosinusitis. Süslü et al.,^11^ using acoustic rhinometry and a saccharine test in patients undergoing septoplasty, noted improvement in nasal obstruction and mucociliary clearance with the use of a hypertonic solution after 20 days. In a randomized study, Satdhabudha et al.^12^ compared the benefits of hypertonic and isotonic solutions with respect to quality of life, nasal score and saccharine test. They concluded that both solutions produced improvement, but the hypertonic solution was significantly superior when used in children with allergic rhinitis for 2 weeks, but there was no difference from isotonic solution after four weeks of use. An *in vitro* study by Min et al.^6^ showed that the ciliary movement diminished and stopped after a few minutes of use of 3% and 7% hypertonic solutions, which they attributed to injury of the nasal epithelium. On the other hand, Kim et al.^13^ observed that the exclusive use of isotonic saline did not cause cell damage compared to hyper- or hypotonic saline, and Viertler et al.^14^ observed less tissue damage with the use of hypertonic saline in nasal mucosae of mice.

The measures of mucociliary function, improvement of nasal inspiratory flow and dosage of inflammatory mediators in different saline concentrations, volumes and conditions have been evaluated, with no definitive consensus.^2^, ^3^, ^7^, ^9^, ^15^, ^16^, ^17^, ^18^ Several authors observed improvement of these parameters with the use of saline solutions as a complementary therapy in patients with chronic nasosinusal disease; however, there is no consensus on the concentration, volume and application method.^2^, ^9^, ^10^, ^15^, ^19^, ^20^

In daily practice, saline solutions for nasal hygiene usually employs normal saline, that is, 0.9% saline, at room temperature, using a positive-pressure dropper or syringe. Sprays, drips, aerosols or nebulizers may also be used, depending on the availability of these resources. All of them have advantages and disadvantages, and cost seems to be a decisive factor in lower income populations. Mello Jr. et al.^2^ draw attention to the fact that the results are not immediate and the adherence to the use of saline may be poor, but the low cost and its few reported adverse effects and the observed clinical improvement justify their use. The improvement in nasal inspiratory flow after the use of saline solutions can be a stimulus for adherence to their continued use, when these products are prescribed.

Among the techniques used to assess improvement in nasal breathing with the use of saline solutions, patient evaluation and self reporting scores have been evaluated.^5^, ^11^, ^12^, ^21^ Those methods are subjective, and in most studies patients were instructed to use saline solutions for several days without instructions for controlling time, frequency, temperature and humidity.^18^, ^22^, ^23^ Rhinomanometry is considered the most reliable technique, but with limited application in field studies.^21^ Another alternative is to use simpler, low-cost portable instruments that measure the nasal inspiratory flow; but it should be borne in mind that these devices are dependent on the capacity to comprehend the procedure and on physical effort. With this modality, while there are no reference values for different populations, the patient can be used as his/her own control.^1^, ^8^, ^24^ The method has a limited role in young children, whose age does not allow the use of inhaled dispensing devices that are dependent on effort.^1^, ^21^, ^23^, ^25^

The aim of this study was to evaluate whether saline solutions at different concentrations and techniques of administration improve nasal inspiratory flow during nasal hygiene practices in healthy children.

## Methods

This series consisted of students aged 8–11 years belonging to an educational institution in a rural city in São Paulo state; a legal guardian for each child was contacted and signed a consent form. This study was approved by the local Ethics Committee under No. CEP 4226-2012.

We first interviewed 20 family members, guardians of children enrolled in an educational institution in a rural city in São Paulo state, in order to learn what measures were used routinely for children to improve nasal breathing in periods of low humidity and heat, or in the presence of rhinosinusal diseases. Based on their responses, we crafted six procedures, as follows:

• Procedure A: no stimulation whatsoever; the child performed only the measurement parameter (nasal inspiratory flow);
• Procedure B: 0.9% saline intranasal – 1 mL in each nostril using a disposable syringe;
• Procedure C: 0.9% saline – 5 mL inhaled by nasal mask through a portable compressed-air device for 5 min;
• Procedure D: 3% saline intranasal – 1 mL in each nostril using a disposable syringe;
• Procedure E: filtered water – 200 mL orally in disposable cup;
• Procedure F: 0.9% saline – 5 mL inhaled by nasal mask through an ultrasonic portable device for 5 min.

The children included in this study were divided randomly into six groups according to the type of initial procedure and in a sequenced manner, with an interval of 48–72 h according to the scheme below.

**Table:** 

Group	Procedure sequence					
1	A	B	C	D	E	F
2	B	C	D	E	F	A
3	C	D	E	F	A	B
4	D	E	F	A	B	C
5	E	F	A	B	C	D
6	F	A	B	C	D	E

Saline solutions and devices were those commonly used by people examined in health services or found in their homes; therefore, the solutions used were not buffered.

For application of inclusion and exclusion criteria, children's parents were questioned about the presence of allergic diseases such as: rhinitis, asthma or atopic dermatitis, previous treatment of rhinitis, nocturnal snoring, supplementary oral breathing, sneezing for a period longer than two days in a row, nasal saline solution use, and how the product was obtained. Children with history of allergy, rhinitis, obstruction, nasal itching or sneezing were classified as rhinitis I. On the other hand, those with a history of rhinitis, sneezing, itchy nose, mouth breathing or snoring, and that had undergone treatment for rhinitis were classified as rhinitis II.

### Exclusion criteria

• Children belonging to age groups under 8 years, in view of the possible difficulties in understanding the inspiratory maneuvers, and children over 11 years, considering the variability in physical development;
• Children with airway infection in the preceding three weeks;
• Children using drugs for allergic respiratory disease in the preceding 6 months;
• Children with history of nasal surgery;
• Children with neuromuscular disease;
• Children with chest deformity.

The study was conducted from February 2012 to November 2013, during school term, on days when children had no tests or physical education classes, in the morning and afternoon, according to their class schedule. The children were called in their classrooms, out of their test period.

For randomization of groups, children's names in each period – morning or afternoon – were listed alphabetically and randomly assigned to one of the six groups. At the beginning of each procedure, the assessment sequence was thoroughly presented to each child.

The efficacy of the various procedures used in this study was evaluated by measuring nasal inspiratory flow, performed by using an In-Check™ nasal inspiratory flow meter device (Clement Clarke International) with an air-cushioned face mask. Peak nasal inspiratory flow was averaged for three determinations, with a one-minute interval between them, and the operator was blinded at each step.

After having undergone one of the procedures and after the three measures of nasal inspiratory flow were averaged, the child was sent back to the classroom, with a recommendation to not run or take water. The time interval between pre- and post-measurement was 30 min.

All procedures and measures of nasal inspiratory airflow were performed by the same professionals. The quantitative results were recorded in an Excel spreadsheet and interpreted the end of the study. Other parameters analyzed were age (in months), weight, height and body mass index. These measurements were transformed to *z*-scores using the Epi-Info™ version 2002 program (nutrition). The environment temperature and relative humidity were also measured. As to the use of saline solutions, the following questions were asked: If the child already made use of these solutions, if this use was restricted to situations of upper respiratory tract infections (URTI), if with exclusive use according to medical prescription. The origin of nasal saline was also investigated: homemade solution, solution provided by the health service (UBS), purchase of pharmaceutical 0.9% saline, and whether the child has already used a pharmaceutical 0.3% hypertonic solution.

The study was conducted in two periods: morning (8:00–10:00 am) and afternoon (1:00–3.00 pm), with simultaneous measurements of temperature and relative humidity, obtained in a local agricultural weather station.

### Statistical analysis

For analysis of the parameters age, gender, weight and height, the *t* test was used for two categories, and ANOVA followed by Tukey test for more than two categories, always considering as significant a result with *p* < 0.05.

## Results

Among the 202 children in the 8–11 year age range enrolled in the school, 129 (56.58%), met the inclusion criteria, of which 5 (3.87%) were excluded during the study. Thus, 124 (96.12%) children completed all steps.

There was no significant difference in age (in months), height, weight, or body mass index.

In the comparison between each procedure, the peak nasal inspiratory flow was significantly increased with 3% *versus* 0.9% saline inhalation with a compressor (*p* = 0.0185) and with an ultrasonic inhaler (*p* = 0.0330). There was no difference between 0.9% *versus* 3% saline by nasal route (*p* = 0.1186). The set of procedures which used saline solutions had a significantly higher peak nasal inspiratory flow, when compared with the use of water, or no stimulation (*p* = 0.0133) (Table 1).

**Table 1:** Comparison of all procedures according to the percentage change in pre- and post-procedure nasal inspiratory flow peak.

Procedure	Mean ± SD	Median (minimum and maximum)
A	5.33 ± 17.09 b	3.94 (−30.43; 75.00)
B	8.44 ± 17.44 ab	5.94 (−28.57; 73.33)
C	6.22 ± 19.06 ab	5.04 (−28.57; 119.35)
D	12.33 ± 21.42 a	7.28 (−26.09; 146.67)
E	5.04 ± 15.16 b	4.81 (−22.22; 52.94)
F	7.26 ± 15.23 ab	5.13 (−29.51; 58.35)

Comparisons made using ANOVA (*p* = 0.0133). Means followed by the same letter do not differ at a 5% level by Tukey test.

There was no significant difference between genders as to overall mean peak inspiratory flow among all procedures (*p* = 0.65331), and individually for each procedure: A (*p* = 0.754); B (*p* = 0.936); C (*p* = 0.328); D (*p* = 0.368); E (*p* = 0.186); and F (*p* = 0.391) (Table 2).

**Table 2:** Distribution of mean values of the percentage change in nasal inspiratory flow peak before and after stimulation applied in 124 children, according to the time of day.

Procedure	Morning	Afternoon	*p*-Value
A	2.68	7.73	0.100
B	5.51	11.10	0.074
C	4.52	7.76	0.346
D	9.54	14.85	0.168
E	3.05	6.84	0.165
F	8.56	6.08	0.368

Values of temperature and relative humidity showed a significant difference, *p* < 0.05, with lower humidity levels in the afternoon and higher temperatures in the same period, for the same procedures. There was no significant difference between mean values for percentage change in peak nasal inspiratory flow according to the procedure sequence.

Most children (54%) showed peak nasal inspiratory flow above the mean for procedure A – no stimulation, with respect to at least one of the dispensing modes and/or concentrations of saline solutions, with no significant difference in gender, period of the day or initial sequence. As to the questionnaire on habitual use of saline solutions by the studied population, it was observed that 80.65% of this population makes use of saline, 50% only in case of flu; 35.49% only when prescribed by a physician; 43.55% buy the product, and the remaining children obtain the product free of charge in health services. The use of hypertonic saline was reported by 4.84% of the participants. 13.71% were classified as rhinitis I, and 7.26% as rhinitis II. There was no significant difference in percentage means of procedures, when comparing the information provided by the questionnaire.

## Discussion

The use of saline solutions has been assessed more frequently in rhinosinusal disease as a measure of hygiene and humidification and as an adjuvant procedure in the maintenance of nasal homeostasis. In this study, as to the set of procedures, there was no significant difference between 0.9% and 3% saline, regardless of the form of administration; however, in the individual comparison among procedures, 3% saline not only was significantly superior *versus* 0.9% saline administered with a syringe; we also observed that 0.9% saline was not superior to other procedures. The concentrations and ways of administering the product make it difficult to assign to one or another method an absolute superiority over all remaining methods, since the responses are individual and therefore may be subject to a large variation.

Topical use of saline solution has been common in the treatment of rhinosinusal disease.^7^, ^15^, ^26^ This is considered an adjuvant therapy, although an improvement in signs and symptoms has been demonstrated with the use of saline alone in less serious situations. In our study, we found that the means of percentage change show significant improvement of peak nasal inspiratory flow with the use of saline solutions. Satdhabudha et al.,^12^ in a randomized and blinded study, compared mucociliary clearance and the total score of nasal symptoms before and 10 min after the use of hypertonic *versus* 0.9% saline in 81 children with allergic rhinitis, and concluded that hypertonic saline produced superior results; but both treatments resulted in improvement in quality of life and symptom scores after 2 weeks of use. Hermelingmeier et al.^10^ in a systematic review, concluded that in cases of allergic rhinitis, the use of saline resulted in improvement in nasal symptoms in 27.6%, reduced the use of drugs in 62.1% and improved quality of life in 27.8% of their patients; however, Achilles et al.^20^ concluded, also in a review article, that it is not possible to standardize this practice for acute rhinosinusitis.

The negative percentage change observed in this study means that patients may experience worsening of nasal flow after the procedure, including the use of saline solutions and, therefore, it does not allow for an universal indication in favor of a given concentration or form of administration. The use of variation of peak nasal inspiratory flow to evaluate an initial obstruction and response to treatment is a practical, simple, and inexpensive method to use in clinical practice, and allows for the selection of the most appropriate saline solutions with respect to concentrations and delivery method appropriate for each patient (Teixeira et al.^27^). Ural et al.^5^ concluded that the use of saline solutions should be selective, and not based on anecdotal evidence.

There is controversy about the exact mechanism of action of saline solutions on nasal mucosa. The use of a saline solution helps reduce nasal symptoms, perhaps by reducing the inflammation of mucosa, but little is known about the effects on human nasal mucosa. Saline solutions are usually well tolerated and present few side effects; additionally these products result in an immediate cleaning up of secretions.

Some authors suggest that, with respect to nasal mucus, 0.9% saline solution would be hypotonic (mucosal osmolality, 390 mOsm/L and solution osmolality, 300 mOsm/L).^14^ The difference of tonicity would cause a deleterious effect to the cells, functioning as a stimulus for proliferation of glands and decrease of ciliary beating. Clinical studies on the benefits of different saline concentrations have conflicting results.^28^, ^29^ In this study, we observed higher means of peak nasal inspiratory flow with the use of saline applied through positive pressure with syringe, but there was no significant difference as to concentration. Heatley et al.^19^ evaluated 150 patients with chronic rhinosinusitis who were asked to use 3% saline and reflexology massage, and concluded that there was improvement of symptoms with both saline and massage. Keojampa et al.^30^ evaluated mucociliary clearance and nasal patency in 22 healthy volunteers, each of them serving as self-control, with the use of 3% or 0.9% saline; and concluded that both saline solutions improved nasal clearance; but with the use of 3% saline the clearance was faster, with no change in nasal patency as measured by acoustic rhinometry. In animal studies, there is also controversy about the effects of different concentrations of saline solutions on the mucosa and ciliary beating.^13^, ^14^, ^31^ Alzérreca et al.,^15^ reviewing the use of antifungal medications, shampoos and solutions in the treatment of rhinosinusal disease, concluded that, to date, there is no definition for ideal pHs, concentrations or temperatures.

The optimal duration of use of these solutions, as well as their daily frequency and form of administration, were not established.^2^, ^3^, ^15^ Wei et al.^17^ found that the use of a saline solution once a day produced an improvement in quality of life after six weeks of use, with 90% of adherence to treatment in 34 children with chronic rhinosinusitis. Jeffe et al.^8^ evaluated the use of 0.9% saline twice a day in 61 children with rhinopathy, and concluded that 86% could tolerate the use of the product, but the adherence was 77%. Although it is not possible to state that patients with worsening nasal inspiratory flow after a given stimulus, as noted in our study, may be less compliant with treatment with the same stimulus, some authors^2^ argue that the adherence may be compromised, considering that the effect on the symptoms may not be immediate.

In the present study, no statistically significant differences were noted in the comparison of 0.9% saline solution in different forms of application, such as positive pressure with syringe, or negative pressure with inhalation using a compression or ultrasonic nebulizer. Mean values were higher with the use of 0.9% saline using positive pressure with a syringe, and this finding can be considered in an empirical initial indication for use of saline in this concentration. In a tomographic evaluation, Olson et al.^23^ concluded that contrast deposition in different sinonasal regions was higher with positive pressure.

We observed that the application of 3% saline with a syringe had a mean peak nasal inspiratory flow superior to 0.9% saline applied by nebulizer, indicating that the concentration and form of administration should be considered together when these products are prescribed, since the concentration and saline deposition may contribute to this result.^23^ There was no statistically significant difference comparing the use of 0.9% and 0.3% saline solutions; but it is worth to consider, in the empirical initial indication (in those cases in which there is no possibility for evaluating the answers to two different concentrations), that 0.3% saline achieved better means. Comparing the use of positive pressure with syringe *versus* nebulization, the temperature of the solution into contact with mucosa could reduce the efficacy of one of these methods. Mean temperatures of saline solutions at room temperature were 5–8 °C higher than those in the compressor nebulizer, and 0.8–1.5 °C higher than those in the ultrasonic nebulizer. The optimal temperature would be that closest to the temperature measured at mucosal level, thereby avoiding responses of vessels and nerve endings to exposure to a lower temperature than that in the environment.

Apparently, the use of saline solutions, even in different concentrations, helps reduce inflammation and inflammatory mediators, cleaning crusts and secretions, softening mucus in patients with rhinopathy, or in daily nasal hygiene.^2^, ^5^, ^16^, ^25^ The use of saline solutions is also used to relieve symptoms in conditions of low relative humidity.^2^, ^16^, ^32^ Krajnik et al.^33^ observed that high humidity can influence the distribution of the aerosols produced by saline nebulization, increasing the particle size. Thus, we must consider the relative humidity and the form of delivery of saline. In our study, we observed that the temperature and humidity were significantly different between morning and afternoon periods, but there was no significant difference in mean peak nasal inspiratory flow in response to procedures, with respect to the time of day. In this study, the children studied live in the same area and were exposed to the same environmental and climatic factors. At a higher humidity condition, it would be expected that the environment water-soluble particles could be deposited on the nasal mucus causing irritation, and that a cleaning procedure with saline could emphasize the improvement of nasal performance, by removing mucus and particles; but no significant difference was noted. Using sensitized mice, Larsen et al.^22^ reported worsening of inflammatory response to exposure to formaldehyde in a low-humidity environment, but not in a high-humidity scenario, suggesting that water-soluble particles are deposited in the upper airways, being imprisoned in mucus. In 18 patients sensitized to pollen and exposed to an atmosphere of 37 °C and a relative humidity greater than 90% before their provocation with the antigen, Baroody et al.^34^ observed reduction of acute inflammatory, neural and vascular responses in patients’ nostrils – a fact that was attributed to the action and local effect on the mucosa.

The use of the percentage change in peak nasal inspiratory flow before and after each procedure reduced potential biases, which would not occur in the household environment, since there would be no way to control whether the child actually used the suggested volume, frequency, and concentration. It also would not be possible to know whether conditions of temperature, humidity and time of day would be the same for everyone. The positive change in measures of not-stimulated flow peak – procedure A – indicates the possibility of improvement in children's performance, perhaps by improving the technique; but all of them were subject to the same conditions (which reduces the risk of interference) and there was no significant difference as to beginning in one or another group of sequence of procedures.

No significant difference in means of procedures was noted when children classified as rhinitis I *versus* those asymptomatic ones were compared. The same was true for those classified as rhinitis II. In children with a history of rhinitis I or II, no significant worsening of response occurred with the use of 3% saline solution, thanks perhaps to the absence of acute or persistent inflammatory processes, since these were exclusion criteria. Alzérreca et al.^15^ found that, for most patients, the benefits outweigh the drawbacks, for instance, a burning sensation observed both with 0.9% and hypertonic solutions. Adappa et al.^35^ argue that hypertonic saline was not superior to 0.9% saline, perhaps by stimulating a neural response, causing vasodilation and a runny nose. Jeffe et al.^8^ noted that 0.9% saline was well tolerated by 83% of children aged 6–12 years, and that their side effects, such as ear pain, local pain, coughing or nausea, were not so important as to stop the study.

In the case of 0.9% saline solutions, there was no difference according to the dispensing method and, therefore, it would be unreasonable that families invest their resources in nebulizer apparatuses for the purpose of cleaning up. The 0.9% saline solution is cheaper, and it is likely that its use, in association with the use of water by mouth to maintain body hydration, is a simple and inexpensive strategy that produces satisfactory effects.

Before this study, few of the children had used saline in higher concentrations, indicating that, in this population, the methods of application and the 0.9% concentration are more traditional. The cost of saline solutions in spray makes it difficult the access to this form of administration.

In this study, sodium absorbed by children using saline solutions ranged from 4% to 15% of their daily needs.

## Conclusion

The results suggest that saline solutions improve the peak of nasal inspiratory flow of most users, and that water intake does not replace the use of these solutions. The mean values obtained suggest that, by nasal route, 3% saline provides better results than 0.9% saline and is significantly superior to 0.9% saline by inhalation.

## Conflicts of interest

The authors declare no conflicts of interest.
